# Risk Factors Associated with Extensively Drug-Resistant Typhoid in an Outbreak Setting of Lyari Town Karachi, Pakistan

**DOI:** 10.4269/ajtmh.21-1323

**Published:** 2022-03-28

**Authors:** Rabab Batool, Sonia Qureshi, Mohammad Tahir Yousafzai, Momin Kazi, Miqdad Ali, Farah Naz Qamar

**Affiliations:** ^1^ Department of Pediatrics and Child Health, Aga Khan University Hospital, Karachi City, Sindh, Pakistan;; ^2^ Center for Child, Adolescent, and Maternal Health, Faculty of Medicine and Health Technology, Tampere University, Tampere, Finland

## Abstract

Typhoid fever is endemic in Pakistan, with high annual incidence rates. An outbreak of extensively drug-resistant typhoid fever that first started in the Hyderabad district of Sindh province in November 2016 immediately spread to the whole province. We conducted an age-matched case–control study to assess the risk factors of typhoid fever in an outbreak setting of Lyari Town, Karachi. We enrolled 82 patients with blood culture-confirmed *Salmonella* typhi between August 2019 to December 2019, 82 age-matched hospital and 164 age-matched community control subjects. In a matched conditional logistic regression model, consumption of meals outside the home more than once per month was associated significantly with developing culture-confirmed typhoid fever compared with no consumption of food outside the home (odds ratio, 4.11). Hygiene of the environment in which food is prepared, practices of adult food handlers, access to clean water, and food legislation play significant roles in the spread of typhoid fever.

Typhoid has remained an important public health concern in impoverished populations of developing countries. Typhoid fever is a commonly observed etiological source of bacteremia in many developing countries of South Asia, Southeast Asia, and sub-Saharan Africa, where it is a major cause of morbidity and mortality among children.^1^ The annual burden of typhoid and paratyphoid fever is estimated at 14.3 million cases and 135,900 deaths worldwide.^2^ In Pakistan, typhoid is the well-known cause of bacteremic illness in children, with rates more than 451.7 per 100,000 among children 2 to 15 years old.^3^ The data from the Surveillance for Enteric Fever in Asia Project, which is a large, multicenter, prospective surveillance study capturing data on the burden of enteric fever and the antimicrobial susceptibility of the isolates in Bangladesh, Nepal, and Pakistan, identified an extremely drug-resistant (XDR) typhoid outbreak in Hyderabad, Pakistan.^3^ First reported in November 2016, the disease spread quickly to the entire Sindh Province and infected thousands of children and adults. The associated *Salmonella* Typhi (*S.* Typhi) H58 strain was resistant to five classes of antimicrobials (chloramphenicol, ampicillin, trimethoprim–sulfamethoxazole, fluoroquinolones, and third-generation cephalosporins) and was labeled an XDR *S. *Typhi. This strain of XDR *S. *Typhi was sensitive only to azithromycin and meropenem.^4^ These drug-resistant strains threatened the role of antibiotics in typhoid control, increased the treatment cost, and inflated significantly the morbidity and mortality rates.^5^

The causative agent of typhoid, *Salmonella* enterica serovar Typhi (*Salmonella* Typhi), is a human host–restricted organism. The risk factors for typhoid include poverty, unhygienic environmental conditions, lack of access to safe drinking water, absence of appropriate sanitation amenities, and hazardous foods.^6^ The disease burden is distributed heterogeneously within different Southeast Asian countries, with the greatest incidence reported in the areas with the most deficient hygiene and sanitation, including urban slums. Transmission is via the fecal–oral route indirectly by the consumption of contaminated water and food with the intestinal content of the cases or the carriers.^7^

Various similar food exposures and hygiene habits have been identified as risk factors for typhoid fever in different countries.^8^ The role of water as a medium for disease transmission has been acknowledged since the late 1800s, and the role of food in disease transmission was recognized centuries ago.^6^ Eating unsafe food, using contaminated utensils, or living in unhygienic conditions favorable for the growth and survival of microorganisms, food cooked at inadequate cooking temperatures, and poor sanitation are the identified risk factors for the spread of the disease in endemic areas. Several studies indicate that street foods indeed have high microbial loads. Unclean water or people handling food who may be carriers can cause food contamination.^8^ Unhygienic food preparation is an indisputable factor contributing to the incidence of foodborne diseases, especially people with inadequate personal hygiene handling food who have had typhoid and exhibit poor self-hygiene.^9^

Even as the typhoid conjugate vaccine poses an auspicious tool for typhoid elimination, targeted water, sanitation, and hygiene interventions are necessary. Comprehensive knowledge of context-specific risk factors for typhoid fever is crucial to appraise non-vaccine control measures.^10^

Previous studies have identified various food and water exposures as risk factors for typhoid fever. Among food items, consumption of any type of ice, including flavored ices, ice cream, and ice cubes, has been identified as a vehicle for the infectious agent. Eating from roadside vendors and consumption of water from outside that is unsafe or untreated have been associated significantly with the potential risk of infection.^8^ In Karachi, eating ice cream, prophylactic antibiotics, consumption of food from roadside vendors during hot weather, and consumption of contaminated water were associated significantly with the risk of typhoid fever.^11^

We proposed a study to identify the factors associated with the risk of typhoid fever in the XDR typhoid outbreak setting of Lyari Town, Karachi. This study targeted the population of children who were 6 months to 15 years old, because children younger than 15 years of age comprise more than 90% of the XDR typhoid cases.^12^

We conducted an age-matched case–control study from August 2019 to December 2019 in the slum area of Lyari Town, Karachi, after a mass immunization campaign with Typbar typhoid conjugate vaccine was conducted in Lyari Town to control the immediate spread of XDR typhoid in children 6 months to 15 years of age. Lyari Town is a peri-urban slum settlement that is sub-divided into 11 union councils. The study was conducted in three key hospitals serving the town, including Lyari General Hospital, Kharadar General Hospital, and Aga Khan Secondary Care Hospital. It was one of the towns in Karachi first hit critically by the XDR typhoid outbreak and was where the outbreak was first identified with the greater number of cases. Age-matched controls were enrolled in the case-to control ratio of 1:1 and 1:2 for case to health facility-based controls and case to community-based controls, respectively.

A case was defined as an individual with blood culture-confirmed *Salmonella* typhoid between the ages of 6 months to 15 years, and a resident of Lyari Town visiting any of the three sentinel hospitals. Control subjects were enrolled if they were at least 7 months of age and ±6 months of the age of a case 6 to 36 months old or ±3 years of the age of a case 3 to 15 years old. Health facility controls were afebrile children who lived in Lyari Town for at least one month, admitted or presented to the emergency department, pediatric ward, orthopedic ward, surgical ward and outpatient department of the same health facility without a history of fever and infection or elective surgery or trauma and found eligible to be enrolled as hospital controls within seven days of the visit date of the case.

Community control subjects were defined as afebrile children living in the neighborhood, Lyari Town, for at least 1 month who had no febrile illness in the 4 weeks before the time of enrollment. Once at the case’s house, the investigator went either to the left or to the right (determined by a coin toss) and interviewed the third neighbor to determine whether a child meeting the selection criteria lived in the household. If no child met the inclusion criteria, then the investigator went to the fourth house and proceeded sequentially until a control subject was enrolled. If there was more than one eligible child in the same household, chits were picked to decide who would be recruited. The trained team member interviewed the parents of all the eligible and enrolled children using a structured questionnaire in the local language. Information was collected on the age, gender, area of residence, and various risk factors for typhoid fever including water, hygiene, and sanitation.

Data were cleaned and imported into STATA version 12 (StataCorp, College Station, TX) for analysis. For univariate analysis differences in demographic, environment and food, and personal hygiene risk factors exposure between cases and control subjects were evaluated. Matched odds ratios, *P* values, and 95% CIs were calculated using maximum likelihood estimates.

We expected that various risk factors might be accountable for disease transmission. Because certain foods and other exposures were closely related, multicollinearity was considered among different variables. We included independent variables with *P* ≤ 0.10 on univariate analysis in a final regression model. Matched conditional logistic regression was performed to deal with the potential confounders and effect modifiers.

The study was approved by the Ethical Review Committee of Aga Khan University Hospital. A child was enrolled into the study if their parent/legal guardian was willing and able to provide informed consent. If the participant was 7 years of age or older, child assent was also sought.

Of 82 cases, 28 were enrolled from Lyari General Hospital, 28 were enrolled from Kharadar General Hospital, and 26 were enrolled from Aga Khan Secondary Care Hospital. A total of 164 community age-matched control subjects and 82 hospital control subjects were enrolled as well.

Participant age ranged from 6 months to 15 years, with a mean age of culture-confirmed typhoid cases of 54.6 months; community control subjects, 58.9 months; and hospital control subjects, 55.1 months. Of the enrolled cases, 68.3% were male, 51.8% of community control subjects were male, and 56.1% of hospital control subjects were male.

Male gender, water bought from local vendors in refillable containers, positive history of family/household member ever in contact with or having had typhoid, frequency of meals eaten outside the home, consumption of food at a restaurant in past 4 weeks, and consumption of cold beverages outside the home in past 4 weeks were associated with typhoid fever in the univariate analysis.

We adjusted for age, gender, source of drinking water, family/household member ever in contact with or having had typhoid, frequency of meals eaten outside the home, consumption of food at a restaurant in past 4 weeks, and consumption of cold beverages outside the home in past 4 weeks in the final multivariable matched conditional logistic regression model. We found that the consumption of meals outside the home more than once per month was associated significantly with the development of culture-confirmed XDR typhoid fever compared with no food consumption outside the home (odds ratio, 4.11; 95% CI, 1.77–9.54; *P* = 0.001) (Table 1).

**Table 1:** Univariate and multivariate analyses using conditional logistic regression for risk factors of *Salmonella* typhi among 82 patients and 246 age-matched hospital and neighborhood control subjects in the extensively drug-resistant typhoid outbreak setting of Lyari Town, Karachi

Risk factors	Control subjects (*n *= 246), *n *(%)	Patients (*n *= 82), *n *(%)	Total (*N* = 328)	Univariate analysis		Multivariate analysis	
				OR (95% CI)	*P* value	aOR (95% CI)	*P* value
Gender							
Male	131 (53.3)	56 (68.3)	187 (57.0)	1.86 (1.10–3.15)	0.020	1.52 (0.88–2.65)	0.132
Female	114 (46.7)	26 (31.7)	141 (43.0)	Ref.	–	Ref.	–
Age, y							
≤ 2	68 (27.6)	24 (29.3)	92 (28.1)	Ref.	–	Ref.	–
2–15	178 (72.4)	58 (70.8)	236 (71.9)	0.57 (0.13–2.46)	0.452	0.44 (0.09–2.18)	0.313
Type of household							
Pucca†	6 (2.4)	1 (2.4)	7 (2.1)	Ref.	–	–	–
Semi-pucca/kaccha‡	80 (97.6)	80 (97.6)	317 (96.6)	1.50 (0.32–6.94)	0.604	–	–
Method of garbage disposal							
Carried away by sweeper/garbage dump	108 (43.9)	34 (41.5)	142 (43.3)	Ref.	–	–	–
Thrown on the street	49 (19.9)	19 (23.2)	68 (20.7)	1.47 (0.61–3.53)	0.390	–	–
Others	89 (36.2)	29 (35.3)	118 (36.0)	1.00 (0.47–2.19)	0.981	–	–
Source of drinking water							
Municipal supply within the house (running water)	162 (65.9)	44 (53.7)	206 (62.8)	Ref.	–	Ref.	–
Water bought from local vendors	63 (25.6)	28 (34.1)	91 (27.7)	1.91 (0.99–3.69)	0.055	1.70 (0.83–3.48)	0.146
community tap/ underground well/others	21 (8.5)	10 (12.2)	31 (9.5)	1.87 (0.75–4.63)	0.178	1.60 (0.60–4.30)	0.351
Method of water purification used							
None	145 (58.9)	47 (57.3)	192 (58.5)	0.93 (0.56–1.56)	0.791	–	–
Boiling/others	101 (41.1)	35 (42.7)	136 (41.5)	Ref.	–	–	–
Source of ice							
Homemade	185 (75.2)	68 (82.9)	253 (77.1)	Ref.	–	–	–
Others (ice depots, neighbors)	61 (24.8)	14 (17.1)	75 (22.9)	0.61 (0.32–1.18)	0.142	–	–
Family/household member ever in contact with or had typhoid							
No	219 (89.0)	68 (82.9)	287 (87.5)	Ref.	–	Ref.	–
Yes	27 (11.0)	14 (17.1)	41 (12.5)	3.00 (1.01–8.90)	0.048	1.62 (0.72–3.65)	0.243
No. of times a day food is prepared at home							
Once	131 (53.3)	37 (45.1)	168 (51.2)	Ref.	–	–	–
Twice	96 (39.0)	39 (47.6)	135 (41.2)	1.48 (0.86–2.54)	0.155	–	–
Thrice	19 (7.7)	6 (7.3)	25 (7.6)	1.13 (0.42–3.05)	0.812	–	–
Food reheated before cooking							
Always	148 (60.2)	53 (64.6)	201 (61.3)	Ref.	–	–	–
Sometimes/never	98 (39.8)	29 (35.4)	127 (38.7)	0.54 (0.24–1.21)	0.139	–	–
Food stored in the refrigerator							
Always	137 (55.7)	45 (54.9)	182 (55.5)	Ref.	–	–	–
Sometimes/never	109 (44.3)	37 (45.1)	146 (44.5)	1.04 (0.62–1.73)	0.896	–	–
Frequency of meals eaten outside the home							
Never	90 (36.6)	14 (17.1)	104 (31.7)	Ref.	–	Ref.	–
Once per month	74 (30.1)	22 (26.8)	96 (29.3)	2.40 (1.05–5.47)	0.037	1.98 (0.82–4.78)	0.130
More than once per month	82 (33.3)	46 (56.1)	128 (39.0)	4.96 (2.29–10.77)	< 0.001	4.11 (1.77–9.54)	0.001
Consumption of food at a restaurant in past 4 weeks	96 (39.0)	42 (51.2)	138 (42.1)	2.16 (1.14–4.11)	0.019	1.29 (0.60–2.80)	0.508
Consumption of cold beverages outside the home in past 4 weeks	102 (41.5)	43 (52.4)	145 (44.2)	2.25 (1.12–4.52)	0.023	2.11 (0.96–4.64)	0.063

aOR = adjusted odds ratio; OR = odds ratio; Ref. = reference.

* Adjusted for age, gender, source of drinking water, main food preparer ever in contact or had typhoid, frequency of meals outside home, consumption of food at restaurant in past 4 weeks, and consumption of cold beverages outside home in past 4 weeks.

† A pucca household is a dwelling that is designed to be solid and permanent, usually built of stone, burnt bricks, cement, concrete, or timber.

‡ A semi-pucca household is a dwelling in which either the roof or the walls but not both are made of substantial materials such as burnt bricks, stone, cement, concrete, or timber. A kaccha household is built of mud bricks; none of roof or walls are made of solid material.

Enteric fever persists as an important public health concern, especially in Asia, the Middle East, Africa, Latin America, and the Pacific islands, where it is endemic.^13^ The dilemma is the emergence and worldwide spread of *Salmonella* Typhi strains resistant to most previously useful antibiotics in these regions, which is subsequently increasing typhoid-related morbidity and mortality.^6^ Epidemic typhoid is different from endemic typhoid. We matched age in our study to evaluate other risk factors of typhoid fever in an outbreak setting.

The association between consumption of meals outside the home and typhoid fever allegedly resulted from contamination of food during preparation and handling, use of unsafe drinking water, poor hygiene practices of adult food handlers, and the use of contaminated utensils used to serve food.

Restaurants in Lyari Town are small, dark, and damp. Some of them have outdoor seating, typically without freezers for storage of food items, and with minimal amenities for washing raw food (chicken and vegetables) and utensils. There are rarely any toilets or handwashing facilities with soap available. The unboiled, unchlorinated water or tap water stored or served in contaminated containers and used in the preparation of food and for drinking purposes at these small restaurants in the impoverished area can cause disease spread.

The results of our study are in agreement with the findings of studies conducted in Karachi^11^ and Indonesia, in which eating from an outside food cabin was reported to be associated significantly with a greater risk of typhoid infection.^14^ Eating out exposes children to a wide variety of food preparers, some of whom can be chronic carriers of *Salmonella* Typhi. Street-side vendors and cheap restaurants buy ice from ice factories, which is made of unclean water not safe for human consumption. *Salmonella* Typhi survives for prolonged periods in ice, and ice used by the street-side vendors and small restaurants is a potential source of infection. Consumption of cold beverages in Santiago^15^ and the Philippines,^16^ and ice in Pakistan was reported previously to be associated with developing typhoid fever.^7^ Moreover, most people with outdoor eating habits often consume ice, chicken, meat, or eggs.^17^

In Pakistan, food stalls and cheap roadside restaurants do not apply for a license or registration, and the food authority does not inspect their food or beverages regularly.^9^ Adult food handlers in slum areas with low literacy rates are not educated on safe food production and personal hygiene precautions to avert enteric infections. They have no idea that they can be chronic carriers of typhoid disease. None of the food department or restaurant owner inspects food preparation and food-handling practices to ensure food safety. Ideally, if food handlers have diarrhea or vomiting or any symptoms of enteric fever, they should go for testing.^12^ In these cases, food handlers should be prohibited from work until 48 hours after the resolution of diarrhea and/or vomiting. In the case of confirmed typhoid fever, public health authorities recommend that the food handlers should not be allowed to work until two stool cultures, taken and tested 2 weeks apart, test negative for *Salmonella* Typhi.^18^^,^^19^

The consumption of meals outside the home increased the risk of typhoid fever significantly in Lyari Town, Karachi. In a typhoid outbreak setting with poor environmental hygiene and sanitation, this high rate indicates wide dissemination of the typhoid pathogen through people involved in food handling, and reveals one of the probable reasons for the typhoid epidemic in Lyari Town.

The introduction of the novel typhoid conjugate vaccine poses an imperative addition to current tools used to prevent typhoid and possibly eradicate the emergence of XDR typhoid.^6^ The vaccination strategies pose prospects for enhancing indirect protection of those at risk through targeted vaccination campaigns and the potential introduction of typhoid vaccines into the national Expanded Program on Immunization.^20^

There are some limitations to this study worth noting. This was a cross-sectional study, and temporal associations could not be ascertained with certainty and there is no way to ascertain whether the factors preceded the outcome. Another limitation of our study is its small sample size. However, the study was carried out in an urban slum of Karachi. There is enough information available to suggest the factors identified in our study are representative of other areas within Pakistan and other urban slums of South Asia. Hence, the results can be generalized for comparable context and study population.

We recommend that the local food regulatory authority should enact legislation for licensing all restaurants to ensure public access to safe food. Pre-employment screening and medical clearance should be made mandatory for all food handlers; screening of food handlers for *Salmonella* should be done periodically.^9^^,^^21^ The quality of food served in the restaurants should be inspected regularly or periodically. Free access to safe drinking water should be ensured in the slum settlements. To assess food handlers’ practices and level of contamination, assessment of the level of personal hygiene practices during work is suggested.^22^ Awareness programs should be organized periodically for food handlers to inspire and educate them about appropriate food-handling practices.^22^^,^^23^ We need to put collective efforts into place to reduce the burden of typhoid endemicity and other enteric diseases in Karachi.

A multipronged strategy is required to make collective efforts at the national, regional, and global levels toward typhoid elimination. It demands continued investments in reducing risks associated with poverty, poor environmental hygiene, inadequate water and sanitation infrastructures, and sustained investments in safe drinking water and food. We should focus on health equity, accessible health care, rapid diagnosis, efficient management and treatment, and targeted immunization strategies within health systems.^6^^,^^24^

## References

[1] Crump JA , 2019. Progress in typhoid fever epidemiology. Clin Infect Dis 68: S4–S9. 30767000 10.1093/cid/ciy846 PMC6376096

[2] Stanaway JD Reiner RC Blacker BF Goldberg EM Khalil IA Troeger CE Andrews JR Bhutta ZA Crump JA Im J , 2019. The global burden of typhoid and paratyphoid fevers: a systematic analysis for the Global Burden of Disease Study 2017. Lancet Infect Dis 19: 369–381. 30792131 10.1016/S1473-3099(18)30685-6 PMC6437314

[3] Ochiai RL Acosta CJ Danovaro-Holliday M Baiqing D Bhattacharya SK Agtini MD Bhutta ZA Canh DG Ali M Shin S , 2008. A study of typhoid fever in five Asian countries: disease burden and implications for controls. Bull World Health Organ 86: 260–268. 18438514 10.2471/BLT.06.039818 PMC2647431

[4] Qamar FN Yousafzai MT Dehraj IF Shakoor S Irfan S Hotwani A Hunzai MJ Thobani RS Rahman N Mehmood J , 2020. Antimicrobial resistance in typhoidal *Salmonella*: surveillance for enteric fever in Asia Project, 2016–2019. Clin Infect Dis 71: S276–S284. 33258934 10.1093/cid/ciaa1323 PMC7705872

[5] Qureshi S Naveed AB Yousafzai MT Ahmad K Ansari S Lohana H Mukhtar A Qamar FN , 2020. Response of extensively drug resistant *Salmonella typhi* to treatment with meropenem and azithromycin, in Pakistan. PLoS Negl Trop Dis 14: e0008682. 33057330 10.1371/journal.pntd.0008682 PMC7561124

[6] Bhutta ZA , 2019. Integrating typhoid fever within the sustainable development goals: pragmatism or Utopia? Clin Infect Dis 68: S34–S41. 30767006 10.1093/cid/ciy957 PMC6376087

[7] Khan MI Ochiai R Soofi S Von-Seidlein L Khan M Sahito S Habib M Puri M Park J You Y , 2012. Risk factors associated with typhoid fever in children aged 2–16 years in Karachi, Pakistan. Epidemiol Infect 140: 665–672. 21676350 10.1017/S0950268811000938

[8] Siddiqui FJ Haider SR Bhutta ZA , 2008. Risk factors for typhoid fever in children in squatter settlements of Karachi: a nested case–control study. J Infect Public Health 1: 113–120. 20701852 10.1016/j.jiph.2008.10.003

[9] Ogah J Adekunle O Adegoke A , 2015. Prevalence of salmonellosis among food handlers and the health implications on the food consumers in Lagos State, Nigeria. J Med Microbiol Diagn 4: 2.

[10] Prasad N Jenkins AP Naucukidi L Rosa V Sahu-Khan A Kama M Jenkins KM Jenney AW Jack SJ Saha D , 2018. Epidemiology and risk factors for typhoid fever in Central Division, Fiji, 2014–2017: a case-control study. PLoS Negl Trop Dis 12: e0006571. 29883448 10.1371/journal.pntd.0006571 PMC6010302

[11] Luby S Faizan M Fisher-Hoch S Syed A Mintz E Bhutta Z McCormick J , 1998. Risk factors for typhoid fever in an endemic setting, Karachi, Pakistan. Epidemiol Infect 120: 129–138. 9593481 10.1017/s0950268897008558 PMC2809381

[12] Qamar FN Yousafzai MT Khaliq A Karim S Junejo A Baig I Rahman N Bhurgry S Afroz H Sami U , 2020. Adverse events following immunization with typhoid conjugate vaccine in an outbreak setting in Hyderabad, Pakistan. Vaccine 38: 3518–3523. 32201138 10.1016/j.vaccine.2020.03.028 PMC7166079

[13] Jong EC , 2012. Enteric fever: typhoid and paratyphoid fever. *Netter’s Infectious Diseases*. WB Saunders, 394–399.

[14] Medise BE Soedjatmiko S Rengganis I Gunardi H Sekartini R Koesno S Satari HI Hadinegoro SR Yang JS Excler J-L , 2019. Six-month follow up of a randomized clinical trial-phase I study in Indonesian adults and children: safety and immunogenicity of *Salmonella typhi* polysaccharide-diphtheria toxoid (Vi-DT) conjugate vaccine. PLoS One 14: e0211784. 30759132 10.1371/journal.pone.0211784 PMC6373931

[15] Black RE Cisneros L Levine MM Banfi A Lobos H Rodriguez H , 1985. Case–control study to identify risk factors for paediatric endemic typhoid fever in Santiago, Chile. *Bulletin of the World Health Organization 63:* 899--904. PMC2536445 3879201

[16] Tinaya-Superable J Castillo M Magboo F Roces M White M Dayrit M Saniel M , 1995. Multidrug resistant *Salmonella typhi* outbreak in metro Manila, Philippines. Southeast Asian J Trop Med Public Health 26: 37–38.

[17] Mouttotou N Ahmad S Kamran Z Koutoulis KC, 2017. Prevalence, risks and antibiotic resistance of *Salmonella* in poultry production chain. Current topics in *Salmonella* and Salmonellosis, Mares M, ed. Rijeka, Croatia: InTech. Chapter 12: 215–234.

[18] Balasegaram S Potter A Grynszpan D Barlow S Behrens R Lighton L Booth L Inamdar L Neal K Nye K , 2012. Guidelines for the public health management of typhoid and paratyphoid in England: practice guidelines from the National Typhoid and Paratyphoid Reference Group. J Infect 65: 197–213. 22634599 10.1016/j.jinf.2012.05.005

[19] Adane M Teka B Gismu Y Halefom G Ademe M , 2018. Food hygiene and safety measures among food handlers in street food shops and food establishments of Dessie Town, Ethiopia: a community-based cross-sectional study. PLoS One 13: e0196919. 29723288 10.1371/journal.pone.0196919 PMC5933796

[20] Sahastrabuddhe S Saluja T , 2019. Overview of the typhoid conjugate vaccine pipeline: current status and future plans. Clin Infect Dis 68: S22–S26. 30767002 10.1093/cid/ciy884 PMC6376107

[21] Lanata CF Tafur C Benavente EL Gotuzzo E Carrillo C , 1990. Detection of *Salmonella typhi* carriers in food handlers by Vi serology in Lima, Peru *Bulletin of the Pan American Health Organization (PAHO) 24: *177--182. 2198973

[22] Thompson CN Kama M Acharya S Bera U Clemens J Crump JA Dawainavesi A Dougan G Edmunds WJ Fox K , 2014. Typhoid fever in Fiji: a reversible plague? Trop Med Int Health 19: 1284–1292. 25066005 10.1111/tmi.12367 PMC4285329

[23] da Vitória AG Oliveira Jd SC de Almeida Pereira LC de Faria CP de São José JFB , 2021. Food safety knowledge, attitudes and practices of food handlers: a cross-sectional study in school kitchens in Espírito Santo, Brazil. BMC Public Health 21: 1–10. 33579231 10.1186/s12889-021-10282-1 PMC7881630

[24] Siddiqui TR Bibi S Mustufa MA Ayaz SM Khan A , 2015. High prevalence of typhoidal *Salmonella enterica* serovars excreting food handlers in Karachi-Pakistan: a probable factor for regional typhoid endemicity. J Health Popul Nutr 33: 1–9. 10.1186/s41043-015-0037-6 PMC5025978 26825058

